# Prevention of post-mastectomy neuropathic pain with memantine: study protocol for a randomized controlled trial

**DOI:** 10.1186/1745-6215-15-331

**Published:** 2014-08-20

**Authors:** Gisèle Pickering, Véronique Morel, Dominique Joly, Christine Villatte, Delphine Roux, Claude Dubray, Bruno Pereira

**Affiliations:** Université d’Auvergne, Pharmacologie Fondamentale et Clinique de la Douleur, Laboratoire de Pharmacologie, Facultés de Médecine/Pharmacie, Clermont Université, F-63000 Clermont-Ferrand, France; Inserm, U1107 Neuro-Dol, F-63001 Clermont-Ferrand, France; CHU Clermont-Ferrand, Inserm CIC 1405, Centre de Pharmacologie Clinique, F-63003 Clermont-Ferrand, France; CHU Clermont-Ferrand, Centre Jean Perrin, Centre de Lutte contre le Cancer, 58 rue Montalembert, F-63000 Clermont-Ferrand, France; CHU de Clermont-Ferrand, Délégation Recherche Clinique & Innovation - Villa annexe IFSI, 58 Rue Montalembert, F-63003 Clermont-Ferrand cedex, France

**Keywords:** Memantine, NMDA receptor, Breast cancer, Mastectomy, Chemotherapy, Neuropathic pain

## Abstract

**Background:**

N-methyl-D-aspartate receptor antagonists are potential therapies for neuropathic pain, and memantine has a good tolerance profile. A preclinical study recently reported that presurgery memantine may prevent neuropathic pain development and cognition dysfunction. Considering the high prevalence of breast cancer and of post-mastectomy neuropathic pain, a clinical trial is carried out to evaluate if memantine may prevent neuropathic pain development and maintain cognitive function and quality of life in cancer patients.

**Methods/Design:**

A randomized clinical trial (NCT01536314) includes 40 women with breast cancer undergoing mastectomy at the Oncology Hospital, Clermont-Ferrand, France. Memantine (5 to 20 mg/day; n = 20) or placebo (n = 20) is administered for 4 weeks starting 2 weeks before surgery. Intensity of pain, cognitive function, quality of life and of sleep, anxiety and depression are evaluated with questionnaires. The primary endpoint is pain intensity on a 0 to 10) numerical scale at 3 months post-mastectomy. Data analysis is performed using mixed models and the tests are two-sided, with a type I error set at α = 0.05.

**Discussion:**

The hypothesis of this translational approach is to confirm in patients the beneficial prophylactic effect of memantine observed in animals. Such a protective action of memantine against neuropathic pain and cognitive dysfunction would greatly improve the quality of life of cancer patients.

**Trial registration:**

ClinicalTrials.gov: NCT01536314 on 16 February 2012

## Background

Medical treatment of neuropathic pain (NP) is still far from being satisfactory, with less than half the patients achieving significant benefit with any pharmacological drug [1]. Several therapies have been developed for the treatment of NP but these methods are not equally effective for all NP patients. N-methyl-D-aspartate receptor (NMDAR) antagonists such as ketamine, memantine or dextromethorphan are potential drugs for NP alleviation [2]. Evidence suggests that NMDAR within the dorsal horn plays an important role in both inflammation and nerve injury-induced central sensitization [3]. Activation of NMDAR is associated with abnormalities in the sensory (peripheral and central) system, resulting in neuronal excitation and abnormal pain manifestations (spontaneous pain, allodynia, hyperalgesia) [4]. Blocking these receptors by antagonists leads to a reduction in pain [5]. A recent review of the literature including 28 randomized clinical trials [2] emphasizes the heterogeneity of doses used, the diversity of pathologies generating neuropathic pain (post-herpetic, post-amputation, diabetes and so forth) and highlights the need to develop clinical trials of good methodological quality with NMDA antagonists. NMDAR antagonists, such as ketamine [6, 7], are prescribed after therapeutic failure with classical treatment but these drugs have severe adverse events that limit their clinical use [8]. Another NMDAR antagonist, memantine, prescribed in Alzheimer’s disease to maintain cognitive function, has minimal side-effects at doses within the therapeutic range, probably because of its specific mechanism of action as it is an uncompetitive antagonist with moderate affinity, strong voltage-dependency and rapid unblocking kinetics [9–11]. Concerning NP alleviation, memantine shows controversial results in human studies [6, 12–15]. We recently demonstrated for the first time in an animal surgical NP model, that memantine prevents the development of NP symptoms and the impairment of spatial memory [16]. With a translational approach, we present a clinical study where memantine (versus placebo) is administered 2 weeks before and 2 weeks after mastectomy in 40 women suffering from breast cancer. Confirmation of preclinical results in this clinical study would constitute a major step for NP prevention by memantine and maintenance of cognition and quality of life in these vulnerable patients.

## Methods/Design

**Figure 1:**
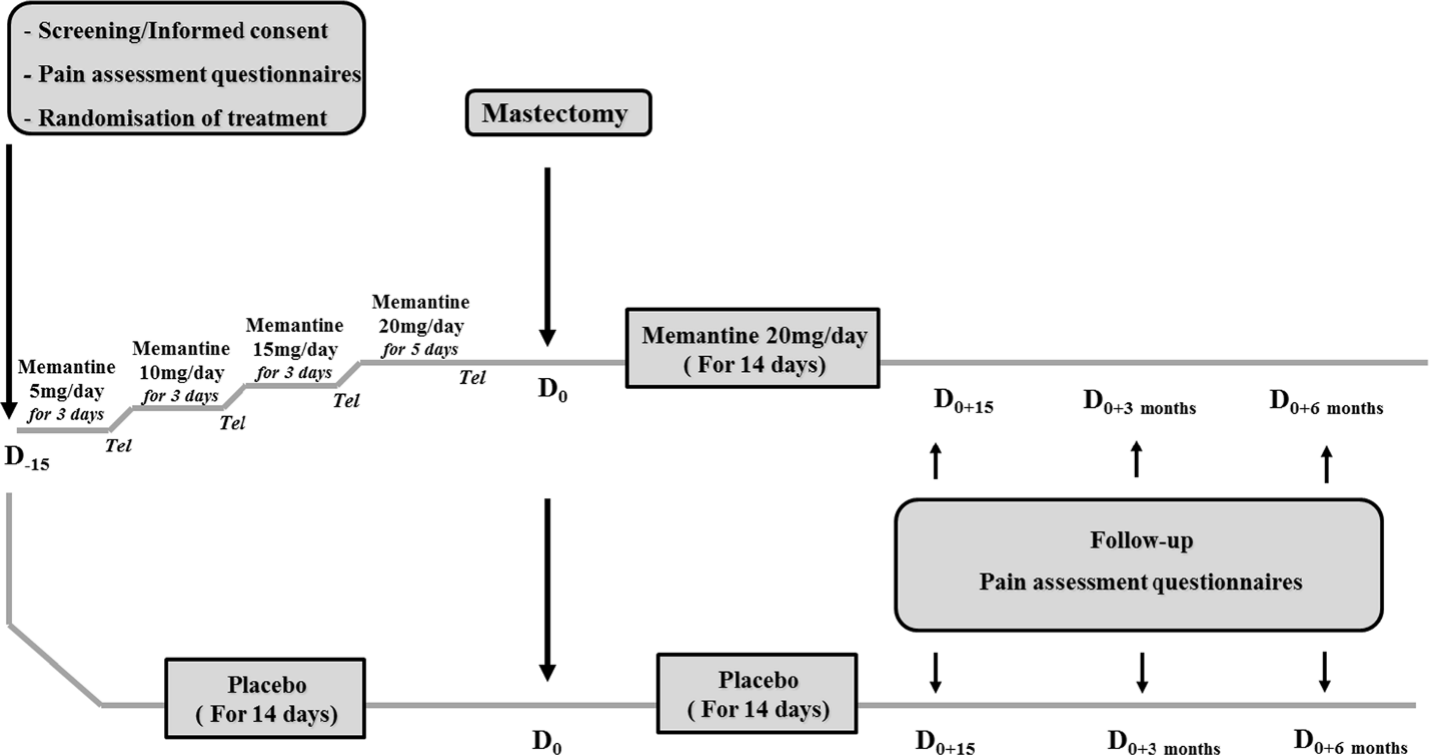
**Study design.**

We are conducting a randomized, placebo-controlled clinical trial in the Oncology Hospital, Clermont-Ferrand, France, in 40 women undergoing total mastectomy for breast cancer. The study has been approved in December 2011 by the regional Ethics committee (CPP Sud-Est, France, number AU917) and registered on 16 February 2012 at “http://www.clinicaltrials.gov” (NCT01536314). Women provide written informed consent prior to their participation in the study during their anesthesiology visit. After baseline assessments (day (D)_−15_) of pain intensity, cognition, quality of life and quality of sleep questionnaires, participants are randomized into two parallel groups: memantine (n = 20) or placebo (n = 20). Memantine or placebo (lactose) is given orally for 4 weeks starting 2 weeks before surgery. Memantine is given in increasing doses: 5 mg/day for 3 days; 10 mg/day for 3 days; 15 mg/day for 3 days and 20 mg/day for 5 days. Endpoints are reassessed 15 days (D_0+15_), 3 months (D_0_ + 3 months) and 6 months (D_0_ + 6 months) post-mastectomy. In order to maintain a good compliance and to verify that women do not develop adverse events, patients are called once a week by phone. A booklet for monitoring is completed daily by the patient for 6 months from the day of surgery. Detailed information on the present study is summarized in Figure 1.

### Eligibility

#### Inclusion criteria

Women are eligible for this study if they are at least 18 years old, with a diagnosis of breast cancer, programmed for mastectomy with or without axillary dissection, able to understand and willing to follow the study protocol.

### Exclusion criteria

Exclusion criteria comprise contraindications for memantine and hypertension, severe cardiac insufficiency or diabetes (Type I and II), alcohol addiction and treatment with specific drugs (amantadine, ketamine, dextromethorphan, L-Dopa, dopaminergic, anticholinergic agonists, barbituric, neuroleptic, IMAO, antispastic agents, dantrolen or baclofen, phenytoin, cimetidine, ranitidine, procainamide, quinidine, quinine, nicotine, hydrochlorothiazide, warfarin). Childbearing age, no use of effective contraceptive method, pregnancy or lactation, involvement in another clinical trial and inability to comply with the requirements of protocol are other exclusion criteria.

### Objectives

The primary objective of this study is to evaluate if memantine administered before and after mastectomy may prevent pain development at 3 months post-mastectomy when compared to the placebo group. The endpoint at 3 months was chosen because chronic pain is usually defined as pain lasting longer than 2 to 3 months [17, 18], and 6 months was included as a secondary endpoint.

The secondary objectives are to estimate at 3 and 6 months post-mastectomy the pain intensity, the analgesic concomitant medications, the impact of treatment (memantine/placebo) on cognitive function, quality of life, sleep, anxiety and depression, the impact of cancer chemotherapy-induced pain and cognitive impairment and the prevalence of phantom breast experience.

### Definition of endpoints and outcome measures

#### Primary endpoint

The primary endpoint is the pain intensity evaluation by numerical scale in memantine and placebo groups at 3 months post-mastectomy. The scale ranges from 0 (no pain) to 10 (maximal tolerable pain).

#### Secondary endpoints

Secondary endpoints are the evaluation of pain at the screening visit, at 2 weeks, 3 months and 6 months after mastectomy (numerical scale, Brief pain Inventory, McGill pain questionnaire), neuropathic pain (Neuropathic Pain in 4 questions, Neuropathic Pain Symptom Inventory), cognition (Trail Making Test, Digit Symbol Substitution Test), sleep (Leeds sleep questionnaire), quality of life (Short-Form-36), and anxiety and depression (Hospital Anxiety and Depression scale). The summary of the different evaluations for a patient is reported in Table 1.

**Table 1:** **Summary of evaluation for a patient**

Visit	Screening/informed consent	Surgery (mastectomy)	Hospital stay	Follow-up	Follow-up	Follow-up
Day of visit	D_0-15_	D_0_	D_0_ to D_0 + 15_	D_0 + 15_	D_0 + 3_ months	D_0 + 6_ months
Filling of questionnaires						
**1- Pain**						
Numerical Scale	+	-	+	+	+	+
DN4	+	-	-	+	+	+
NPSI	-	-	-	+	+	+
BPI	-	-	-	-	+	+
McGill pain questionnaire	-	-	-	-	+	+
**2- Cognition**						
Trail Making Test	+	-	-	+	+	+
Digit Symbol Substitution Test	+	-	-	+	+	+
**3- Quality of life**						
SF-36	+	-	-	+	+	+
**4- Sleep**						
Leeds questionnaire	+	-	-	+	+	+
**5- Anxiety/Depression**						
HAD scale	+	-	-	-	+	+
**6- Concomitant medication recording**						
Treatments (analgesics, antidepressants, etc.)	+	+	+	+	+	+

+, Questionnaire performed; -, questionnaire not carried out; BPI, Brief Pain Inventory; DN4, Neuropathic Pain in 4 questions; HAD scale, Hospital Anxiety and Depression Scale; NPSI, Neuropathic Pain Symptom Inventory; SF-36, Short-Form-36.

##### Brief Pain Inventory

This self-administered questionnaire provides information on the intensity of pain, along with the degree to which the pain interferes with the everyday functioning of life including: mood, walking, general activity, relations with others, sleep, enjoyment of life [19].

##### McGill pain questionnaire

This questionnaire allows the patient to describe pain experienced during the last 48 hours [20]. It has fifty eight qualifiers divided into sixteen items (A to P). Each qualifier is rated from 0 to 4, where 0 = absent, 1 = low, 2 = moderate, 3 = strong, 4 = very strong. The score is divided between two subclasses: sensory subclass (items A to I) and emotional subclass (items J to P).

##### Neuropathic pain: “Neuropathic Pain in 4 questions”

Neuropathic pain in four questions is a clinical tool for the diagnosis of neuropathic pain [21]. This questionnaire has four questions divided into 10 items related to the interview (that is, symptoms) and to the sensory examination (that is, signs). The investigator asks and examines the patient and notes a response “no” or “yes” for each item: “yes” is scored as “1” and “no” is scored as “0”. The sum of scores gives the total score of the patient (out of 10). Neuropathic pain in four questions is considered as positive if the patient obtains a score of 4/10.

##### Neuropathic Pain Symptom Inventory

Neuropathic Pain Symptom Inventory is a self-questionnaire and includes 10 pain descriptors [22]. Intensity is rated on 0 to 10 numerical scales and two temporal items are designed to assess spontaneous ongoing pain duration and the number of pain paroxysms over 24 hours. This questionnaire discriminates five distinct clinically relevant dimensions: spontaneous burning pain, spontaneous deep pain, paroxysmal pain, evoked pain, and paresthesia/dysesthesia.

##### Trail Making Test

This non-verbal cognitive test assesses the ability of speed, executive functions, attention, concentration, and visual perceptual speed [23, 24]. The test takes place in two parts: in Part A, circles are numbered from 1 to 25 and the patient must connect with lines the numbers in ascending order (1-2-3-4, and so forth); in Part B, the circles contain numbers from 1 to 13 and letters from A to L, the patient must connect the circles with lines but alternating numbers and letters (1A-2B -3C, and so forth). The patient must connect the circles as quickly as possible for both parts of the test, without lifting the pen from the paper. The Trail Making Test B additionally provides an estimate of mental flexibility.

##### Digit symbol substitution test

The digit symbol substitution test is a neuropsychological, non-verbal test, which assesses cognitive deficit and brain damage associated with aging and/or depression [25]. It also evaluates learning ability, concentration and attention. It consists of combining pairs of symbols and numbers as quickly as possible and the score is the correct number of symbols in the time allowed (for example, 90 or 120 seconds).

##### Short-Form 36

The Short-Form-36 is a questionnaire evaluating the quality of life of patients [26, 27]. It is a multidimensional scale that assesses the health and quality of life. This scale can be performed in self- or hetero-questionnaire with 36 items including nine dimensions: physical function, role physical, bodily pain, general health, vitality, social functioning, role emotional, mental health and health thinking.

##### Leeds sleep questionnaire

The Leeds sleep evaluation [28, 29] questionnaire is a standardized self-administered questionnaire composed of ten visual analogue scales that relate to four aspects of sleep efficiency: quality of sleep, getting to sleep (visual scales 1, 2 and 3); sleep quality (visual scales 4 and 5); awakening from sleep (visual scales 6, 7 and 8); and behavior following wakefulness (visual scales 9 and 10).

##### Hospital Anxiety and Depression scale (HAD)

The Hospital Anxiety and Depression scale is a self-administered questionnaire in 14 items completed by the patient [30]. It is used to determine the levels of anxiety and depression. Seven of the items relate to anxiety and seven relate to depression.

### Randomization, allocation concealment and blinding

Women with breast cancer are informed by their anesthetist 2 to 3 weeks before mastectomy. On the day of the visit, inclusion and exclusion criteria are verified and written informed consent is obtained by the physician. After clinical examination and pain evaluation, the patient fills in the questionnaires. A clinical nurse independent from the protocol obtains the randomization number from the hospital pharmacy and the patient is then randomized in the memantine or placebo group. Treatment allocation follows a predetermined randomization list and is generated using random blocks. Memantine and placebo treatments are packed in similar blisters covered with an identical label indicating batch number, expiry date and sponsor code with no indication of the name of the drug. The nurse gives the treatment to the patient in an independent room after questionnaires have been filled. In order to maintain blinding, the consent physician (who evaluates pain, the main endpoint of the study) cannot guess allocation at any time and does not see the patient again before they leave hospital.

### Sample size

The number of subjects required is 40 chronic pain patients (20 in each group). The minimum δ difference in numerical scale pain between memantine and placebo groups at 3 months is estimated at 1.6 and σ standard deviation at 1.5, estimated from published data of the literature [31, 32], with α = 0.05 two-sided situation and β = 0.10.

### Statistical analysis

Statistical analyses will be performed with Stata software (version 13, StataCorp, College Station, USA). Concerning the primary objective, comparison between the randomized groups will be performed using an analysis of covariance with baseline score as a covariate [33]. The correlation between baseline and follow-up scores was also proposed. For other secondary parameters, the comparisons between the randomized groups will be performed using the Student test or the Mann–Whitney test (if the conditions for validity of the Student test are not met, normality will be verified by the Shapiro-Wilk and homoscedasticity by the Fisher-Snedecor test). To study the evolution of the main endpoint (numerical scale pain), data analyses will be performed using mixed models which allow us to consider, on the one hand, time, group and interaction time versus group as fixed effects, and, on the other hand, the within- and between-subject variability in order to visualise the assumption of a difference at 2 months between the two randomized groups that increases up to 3 months and stabilizes at 6 months. Residual normality will be checked for all considered models. When appropriate, anticancer chemotherapy (yes/no) will be studied as a fixed effect in these models before considering subgroup analyses. The comparison between the treatment groups will be performed systematically: (1) without adjustment; and (2) by adjusting other factors whose repartition could be, despite the randomization, unbalanced between the treatment groups. The tests will be two-sided, with a type I error set at α = 0.05. A sensitivity analysis of missing data will be performed to ensure the pertinence of the longitudinal data (MAR (Missing at random) or MCAR (Missing completely at random)). In order to assess the problem caused by missing longitudinal data at 6 months, estimation methods developed by Verbeke and colleagues [34] will be proposed.

## Discussion

NP is difficult to treat and NMDAR antagonists, such as ketamine, dextromethorphan or memantine [5, 6, 35], are potential drugs for persistent pain. Memantine has shown its efficacy in some studies [15, 36] and presents the advantage of having less adverse effects than ketamine [8–11]. We have shown in a surgical pain model that memantine prevents NP symptoms such as tactile allodynia and mechanical hyperalgesia when administered a few days before surgery [16]. These findings needed to be confirmed in patients during the post-operative period. Mastectomy is known to generate NP in 23% patients at 3 months post-surgery [37], 42% at 5 years [38] and 37% at 9 years post-mastectomy [39]. Cancer patients undergoing surgery may also develop NP associated with cancer chemotherapy. It is well known that 25% to 50% of patients treated with chemotherapeutic agents such as taxane, vinca alkaloid and platinum classes develop peripheral neuropathy syndrome. Patients with chemotherapy-induced NP display a set of neuropathic pain symptoms that are characterized by stinging, tingling, numbness, changes in sensitivity, burning sensations or electric shocks [40]. The incidence of chemotherapy-induced NP depends on the type of chemotherapeutic agents, the dose administered and the cumulative dose.

This trial of prophylactic memantine given long before surgery in cancer patients aims to evaluate if memantine may diminish overall post-surgery pain and prevent NP development. It aims also to evaluate the concomitant impact on cognition and quality of life. We showed in animals that pre-surgery memantine prevents cognition impairment [16]. Breast cancer as well as chronic pain induced by chemotherapy may be associated with cognitive dysfunction [41] and impairment of health-related quality of life, quality of sleep, anxiety and/or depression [42]. Indeed, cognitive impairment may be due to the diagnosis of the pathology, to surgery [41] and also to chemotherapy, as cognitive deficit is described in 20-30% to 75% of women having chemotherapy-induced NP [43, 44].

Given that mastectomy with or without chemotherapy can induce NP and cognitive and emotional impairment, these complications represent a real burden for women with breast cancer, and global care is a priority to improve their well-being. If our study confirms preclinical results, memantine given before mastectomy could be a new prophylactic strategy to counteract NP development. It would provide a preventive therapeutic innovation to decrease the incidence of post-operative and chemotherapy-induced NP, cognitive impairment and quality of life impairment and comorbidities that generally accompany breast cancer pathology.

## Trial status

Recruitment started in March 2012.

## Abbreviations

NMDAR: N-methyl-D-aspartate receptor
NP: neuropathic pain.
